# CIITA-Linked Antigen Presentation Is Differentially Associated with Interferon and Inflammatory Programs in Stimulated Human Dendritic Cells

**DOI:** 10.3390/biology15080636

**Published:** 2026-04-17

**Authors:** Vural Yilmaz

**Affiliations:** Biotechnology Research Center (BRC), Cyprus International University (CIU), Via Mersin 10, Nicosia 99258, Northern Cyprus, Türkiye; vyilmaz@ciu.edu.tr; Tel.: +90-392-671-11-11 (ext. 2617)

**Keywords:** dendritic cells, interferon-stimulated genes, NF-κB signaling, CIITA, MHC class II, gene expression modules

## Abstract

Dendritic cells (DCs) integrate innate immune sensing with adaptive immune priming through coordinated transcriptional programs that regulate antiviral defense, inflammatory signaling, and antigen presentation. However, the hierarchical organization and interdependence of these pathways following stimulation remain incompletely defined. Here, we performed an in silico re-analysis with full reproducibility of publicly available RNA-sequencing data (GSE108526) to characterize the temporal architecture and associations of immune transcriptional modules in human dendritic cells at 6 h and 16 h following innate immune activation. Principal component analysis revealed stimulation status as the dominant source of transcriptomic variance. Differential expression analysis confirmed robust induction of interferon-stimulated genes (ISGs) alongside modulation of inflammatory mediators and antigen presentation-associated genes. Module-level quantification showed that interferon signaling constituted the primary early transcriptional axis, whereas inflammatory cytokine programs displayed moderate induction and antigen presentation-associated genes exhibited distinct temporal dynamics. Association analysis demonstrated strong relationships between CIITA and downstream MHC class II genes, supporting coordinated antigen presentation regulation, while relationships between interferon and inflammatory modules were positive but non-proportional, indicating partial modular independence. Collectively, these findings reveal a structured yet non-uniform transcriptional organization in stimulated human dendritic cells, characterized by dominant interferon responses accompanied by context-dependent inflammatory activation and differentially associated antigen presentation programs. This integrative framework provides a reproducible systems-level approach for dissecting immune transcriptional architecture in human dendritic cell activation.

## Simple Summary

Dendritic cells coordinate antiviral defense and antigen presentation through complex gene expression programs. However, how these programs relate to one another during immune activation remains incompletely understood. In this study, we reanalyzed publicly available transcriptomic data to examine the relationships between interferon responses, inflammatory signaling, and antigen presentation pathways in human dendritic cells following TLR7 stimulation. Our results show that interferon-stimulated genes dominate early responses, while inflammatory programs display more dynamic, time-dependent behavior. In contrast, antigen presentation-associated genes exhibit a distinct and more stable expression pattern. These findings highlight that major immune transcriptional programs are differentially associated rather than uniformly regulated, providing a more nuanced view of dendritic cell activation.

## 1. Introduction

Dendritic cells (DCs) are professional antigen-presenting cells that orchestrate the transition from innate sensing to adaptive immune activation by integrating pathogen recognition, cytokine signaling, and antigen-processing machinery into coordinated transcriptional programs [1,2]. Upon activation through pattern-recognition receptors (PRRs), DCs undergo rapid functional maturation characterized by upregulation of inflammatory cytokines, co-stimulatory molecules, and major histocompatibility complex (MHC) pathways that collectively determine the magnitude and quality of T-cell priming [2,3,4]. Human monocyte-derived dendritic cells (moDCs) remain a widely used experimental model for dissecting these mechanisms and provide a tractable system for investigating transcriptional remodeling upon innate immune activation [5].

Engagement of innate immune receptors initiates a tightly regulated cascade involving interferon regulatory factors (IRFs) and NF-κB family transcription factors, which together organize antiviral and inflammatory gene expression [6,7,8]. Type I interferon (IFN-I) production is driven primarily by IRF3 and IRF7 activation downstream of nucleic acid-sensing pathways and leads to autocrine and paracrine amplification via the JAK–STAT pathway, culminating in induction of interferon-stimulated genes (ISGs) such as ISG15, IFIT family members, MX1, and OAS1 [6,9]. These ISGs execute antiviral effector functions and modulate antigen presentation, metabolic state, and cytokine sensitivity. Importantly, IFN signaling establishes feed-forward transcriptional networks that can sustain or reshape the immune response beyond the initial stimulus [9,10].

Concurrently, NF-κB signaling—activated via canonical and non-canonical pathways—drives transcription of inflammatory mediators including IL1B, TNF, IL6, and CXCL8 [7,11]. The NF-κB family (RelA/p65, c-Rel, RelB, p50, p52) regulates overlapping but distinct transcriptional programs, and the balance between subunits influences inflammatory amplitude, DC survival, and maturation [7,12]. Notably, cross-regulatory interactions between NF-κB and IRF signaling pathways allow for coordinated yet non-identical regulation of antiviral and inflammatory genes, resulting in modular transcriptional architectures rather than uniform activation [8,13].

A central functional hallmark of activated DCs is the enhancement of antigen presentation capacity through MHC class II pathways that enable CD4^+^ T-cell priming. Transcription of MHC-II genes is controlled by the class II transactivator (CIITA), a non-DNA-binding master regulator that coordinates expression of HLA-DRA, HLA-DRB1, HLA-DPA1, HLA-DPB1, and associated accessory components including CD74 [14,15]. CIITA activity itself is regulated by multiple upstream signals, including IFN-γ and inflammatory pathways, as well as epigenetic modifications and promoter-specific control mechanisms [14,16]. While interferon signaling can enhance CIITA expression, antigen presentation programs may exhibit delayed or differential kinetics relative to early antiviral gene induction, suggesting that MHC-II regulation may represent a secondary or hierarchically downstream event in certain activation contexts.

Recent transcriptomic studies have highlighted that immune activation is organized into partially independent gene modules whose coordination varies across stimuli and time [10,17]. Systems-level analyses demonstrate that interferon-dominant states, inflammatory-dominant states, and antigen-presentation-dominant states can emerge as distinct transcriptional axes rather than components of a single unified program [13,17]. However, quantitative characterization of inter-module relationships, particularly the relationship between CIITA-dependent antigen presentation and interferon/inflammatory gene programs, remains limited in human DC models.

Public RNA-sequencing datasets provide an opportunity to interrogate these relationships through reproducible computational re-analysis. By integrating global variance assessment, transcriptome-wide differential expression, predefined immune module quantification, and correlation-based association analyses, one can move beyond descriptive gene lists toward structured evaluation of transcriptional architecture. Such an approach is particularly valuable for clarifying whether antigen presentation programs scale proportionally with interferon activation, whether inflammatory and antiviral axes remain tightly linked, and how these relationships evolve over time following stimulation.

In this study, we performed a structured in silico re-analysis of human dendritic cell RNA-seq dataset GSE108526 to characterize the temporal organization and associations of interferon, inflammatory, and antigen-presentation transcriptional programs at early (6 h) and later (16 h) time points following innate immune activation. We combined principal component analysis, transcriptome-wide differential expression, curated immune module visualization, quantitative module scoring, and CIITA-centered association analysis to delineate how these pathways relate to one another. Rather than identifying individual pathway activation in isolation, this study provides a quantitative systems-level comparison of major immune transcriptional modules, revealing differences in magnitude, temporal dynamics, and inter-module relationships. This integrative framework reveals the degree to which immune transcriptional programs are coordinated, hierarchically organized, or partially independent in stimulated human dendritic cells.

## 2. Materials and Methods

### 2.1. Dataset Acquisition

Publicly available RNA-sequencing data were obtained from the NCBI Gene Expression Omnibus (GEO) under accession GSE108526. The dataset consists of human dendritic cells subjected to innate immune stimulation, with samples collected at 6 h and 16 h alongside matched unstimulated controls.

Human monocyte-derived dendritic cells were stimulated with the Toll-like receptor 7 (TLR7) agonist resiquimod (R848) or treated with vehicle control, with samples collected at 6 h and 16 h post-stimulation. Each condition included three independent biological replicates (*n* = 3 per group), resulting in a balanced experimental design suitable for comparative transcriptomic analysis. Cells were derived from human donors and differentiated in vitro prior to stimulation. Detailed experimental parameters, including stimulant concentration and culture conditions, were obtained from the original GEO submission (GSE108526) and associated publication, and were consistent across all analyzed samples.

Normalized gene-level expression values (TPM) and associated metadata files were downloaded directly from GEO. Gene annotation corresponding to the human reference genome GRCh38.p13 was retrieved from the provided annotation file accompanying the series.

To ensure a focused and biologically interpretable analysis, only R848-stimulated and matched control samples were included. Additional stimulation conditions present in the dataset (pU/UC, xRNA, noRNA) were excluded to avoid introducing stimulus-specific heterogeneity unrelated to canonical TLR7-driven activation.

No new human or animal experiments were conducted; all analyses were performed on publicly available, de-identified transcriptomic data.

### 2.2. Gene Annotation and Expression Matrix Processing

The TPM expression matrix was indexed by NCBI GeneID identifiers. To enable biologically interpretable gene-level analyses, GeneID entries were mapped to official gene symbols using the GRCh38.p13 annotation table. Entries lacking valid gene symbols were removed. When multiple GeneID entries corresponded to the same gene symbol, expression values were averaged to obtain a single gene-level measurement per sample. For visualization and module-level analyses, expression values were variance-stabilized using log transformation: log_2_(TPM + 1).

This transformation reduces skewness and mitigates the influence of highly expressed genes while preserving relative differences across samples. It should be noted that while log-transformed TPM values were used for visualization and module scoring, differential expression analysis was performed separately on raw count data using the DESeq2 framework (1.44.0.), as described below.

### 2.3. Principal Component Analysis

The principal component analysis (PCA) was performed on the complete log_2_-transformed gene expression matrix after gene annotation filtering. PCA was conducted to evaluate global transcriptional variance and to determine clustering patterns according to stimulation status and time point.

The proportion of variance explained by each principal component was calculated and used to assess the relative contribution of biological factors to overall transcriptomic variability.

### 2.4. Differential Expression Analysis

To identify genes differentially expressed following stimulation, we compared stimulated samples to matched control samples at each time point. Differential expression analysis was performed using the DESeq2 framework, which models count data using a negative binomial distribution and provides robust estimation of dispersion and variance across biological replicates. Raw count data were used as input, and size-factor normalization was applied to account for differences in sequencing depth between samples.

For each gene, log_2_ fold changes were calculated between stimulated and control groups at each time point. Statistical significance was assessed using the Wald test as implemented in DESeq2. Resulting *p*-values were adjusted for multiple testing using the Benjamini–Hochberg false discovery rate (FDR) correction.

Genes were considered differentially expressed if they satisfied both criteria: |log_2_ fold change| ≥ 1 and adjusted *p*-value (FDR) < 0.05. The fold-change threshold was selected to capture biologically meaningful expression differences, while the FDR threshold controls for multiple testing. Differential expression results were visualized using volcano plots displaying log_2_ fold change against –log_10_(FDR).

### 2.5. Immune Gene Module Definition

Three biologically defined gene modules were constructed based on established immunological functions: (1) Antigen Presentation/MHC Class II Module: CIITA, CD74, HLA-DRA, HLA-DRB1, HLA-DPA1, HLA-DPB1; (2) Interferon-Stimulated Gene (ISG) Module: ISG15, IFIT1, IFIT3, MX1, OAS1, IRF7; and (3) Inflammatory Cytokine Module: IL1B, TNF, IL6, CXCL8, NFKBIA.

Gene sets were curated based on well-established canonical pathway components and selected to represent key functional axes of dendritic cell activation rather than to provide exhaustive pathway coverage. A targeted gene selection strategy was adopted to enable biologically interpretable module-level comparisons across conditions while minimizing noise introduced by low-confidence or context-dependent genes.

Importantly, gene sets were predefined based on prior biological knowledge and were not modified after dataset inspection to avoid analytical bias and preserve the reproducibility of the analysis.

### 2.6. Heatmap Normalization

For heatmap visualization, gene-wise Z-scores were computed across all samples:

$$Z=\frac{x-\mu }{\sigma }$$

where x represents the log_2_(TPM + 1) expression of a given gene in a sample, and μ and σ denote the mean and standard deviation across samples.

This approach facilitates visualization of relative upregulation and downregulation independent of absolute expression levels.

### 2.7. Module Score Calculation

Module scores were computed per sample as the arithmetic mean of log_2_(TPM + 1) expression across genes within each module:

$$\mathrm{Module}\;\mathrm{Score}=\frac{1}{n}\sum_{i=1}^{n}\log_{2}(TPM_{i}+1)$$

To enable biologically interpretable comparisons across conditions, module scores were expressed as Δ values relative to the Control 6 h baseline:

$$\Delta \mathrm{Module}\;\mathrm{Score}={\mathrm{Module}\;\mathrm{Score}}_{sample}-{\mathrm{Module}\;\mathrm{Score}}_{Control\;6h}$$

Baseline values were calculated as the mean of all control samples at 6 h, providing a consistent reference point for evaluating relative transcriptional changes across conditions and time points. Group-level summaries are presented as mean ± standard error of the mean (SEM).

### 2.8. Correlation and Association Analysis

To assess relationships between immune transcriptional programs, association analyses were performed using Δ-transformed expression values. For evaluation of CIITA-dependent MHC-II regulation, the MHC-II module score was recalculated excluding CIITA to prevent artificial self-correlation.

Associations between modules were quantified using Pearson correlation coefficients (r) calculated across all samples. Statistical significance was determined using two-sided Pearson correlation testing.

To provide a more robust statistical interpretation, 95% confidence intervals (CI) for correlation coefficients were calculated. In addition, multiple testing correction was considered when evaluating associations across multiple module comparisons. To further evaluate the internal consistency of predefined gene modules, within-module co-expression analysis was performed using Pearson correlation coefficients calculated between all gene pairs within each module. Correlation matrices were visualized as heatmaps (Supplementary Figure S2). These analyses are intended to capture patterns of co-expression and statistical association rather than to imply causal or hierarchical regulatory relationships between pathways.

### 2.9. Statistical and Computational Environment

All analyses were performed in Python 3.11.6 using the following packages: pandas (2.2.2), NumPy (1.26.4), SciPy (1.13.1), and Matplotlib (3.8.4).

All statistical tests were two-sided, and adjusted *p*-values were calculated using the Benjamini–Hochberg false discovery rate (FDR) correction method.

Additional validation analyses, including DESeq2-based differential expression results, are provided in the Supplementary Materials (Supplementary Figure S1).

### 2.10. Reproducibility and Supplementary Computational Package

To ensure transparency and full computational reproducibility, all scripts used for data processing, statistical analysis, module scoring, and figure generation are provided as Supplementary Material accompanying this manuscript (Supplementary Data S1: Reproducibility and computational package). The package includes fully annotated Python and R scripts, processed intermediate data tables, figure-generation scripts, and dependency specifications required to reproduce the analysis environment. Python (version 3.11.6) was used for data preprocessing, visualization, and module-level analyses, while R (version 4.4.3) and the DESeq2 framework were used for differential expression analysis and statistical validation. All analyses can be reproduced using the publicly available dataset together with the provided code base, enabling complete traceability from raw data to final figures.

## 3. Results

### 3.1. Global Transcriptional Remodeling Following Innate Immune Stimulation

**Figure 1:**
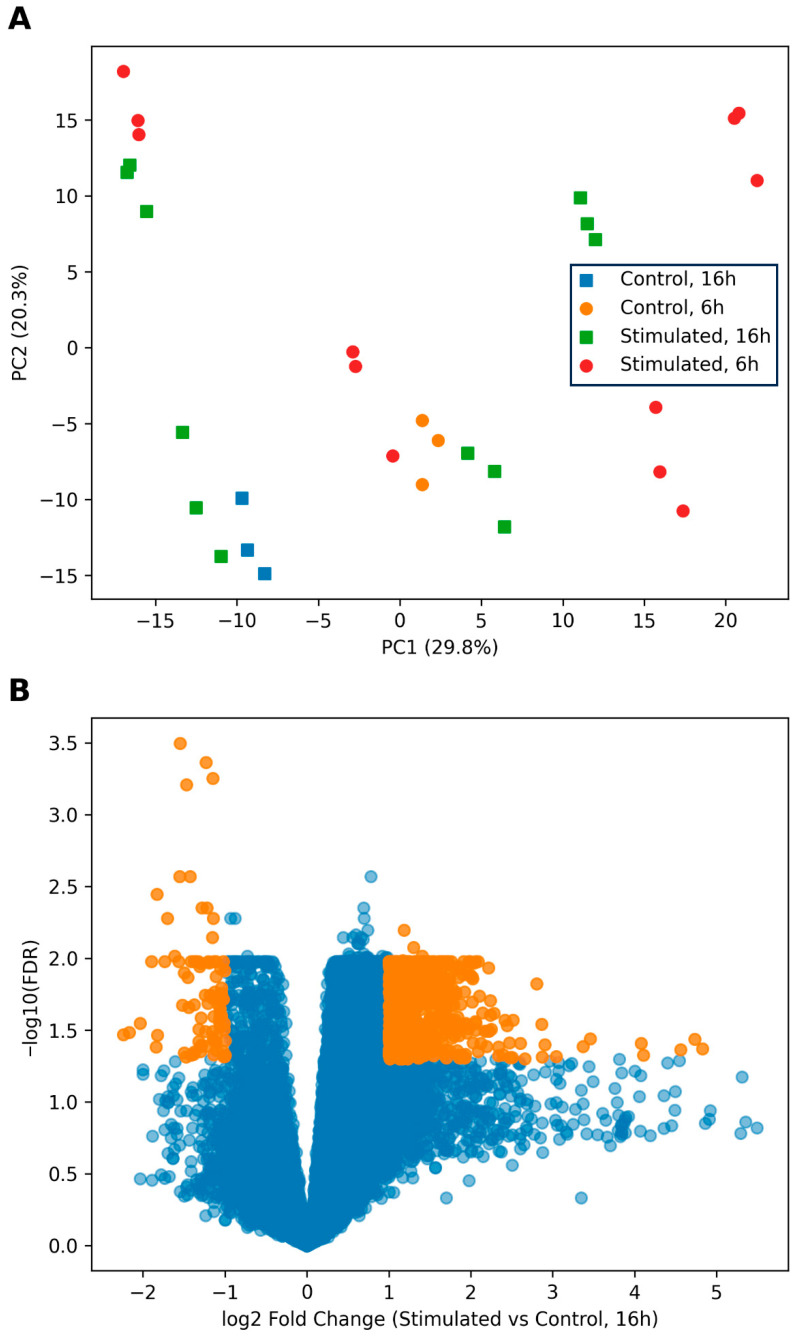
Global transcriptomic remodeling of human dendritic cells following innate immune stimulation. (**A**) Principal component analysis (PCA) of transcriptomic profiles from human monocyte-derived dendritic cells in dataset GSE108526. Analysis was performed using log2-transformed TPM values with gene-wise z-score normalization. Samples are colored according to condition (Control vs. Stimulated) and shaped according to time point (6 h vs. 16 h). PC1 (29.8% of variance) primarily separates samples by stimulation status, while PC2 (20.3% of variance) captures temporal differences between early and late responses, indicating robust and structured transcriptional reprogramming following innate immune activation. (**B**) Volcano plot showing differential gene expression between stimulated and control dendritic cells at the 16 h time point. Differential expression analysis was performed using the DESeq2 framework on raw count data, with statistical significance assessed using the Wald test and false discovery rate (FDR) correction. The *x*-axis represents log_2_ fold change (stimulated vs. control), and the *y*-axis represents −log_10_(FDR). Genes passing both significance (FDR < 0.05) and effect size thresholds (|log_2_ fold change| > 1) are highlighted. Selected immune-relevant genes, including those associated with antigen presentation (CIITA, HLA-DRA, CD74), interferon signaling (ISG15, IFIT1), and inflammation (IL1B, TNF), are annotated to illustrate representative transcriptional responses.

To determine the overall impact of stimulation on dendritic cell transcriptional programs, we first performed principal component analysis (PCA) on log_2_-transformed TPM values (Figure 1A). Samples segregated primarily according to stimulation status along the first principal component (PC1, explaining ~29.8% of total variance), indicating that innate immune activation constitutes the dominant source of transcriptomic variability. Temporal effects were observed along the second principal component (PC2, explaining ~20.3% of total variance), with partial separation between 6 h and 16 h samples within each condition, suggesting gradual transcriptional reprogramming within each condition. While PCA provides an unsupervised visualization of global transcriptional structure, formal statistical testing of group separation (e.g., PERMANOVA) was not performed, as differential expression and module-level analyses were used to quantitatively assess condition- and time-dependent effects. The clear separation of stimulated and control samples in PCA is consistent with the extensive differential gene expression observed in subsequent analyses.

Differential expression analysis further confirmed substantial transcriptional remodeling following stimulation (Figure 1B). The volcano plot demonstrates widespread gene induction and repression, with numerous genes surpassing both fold-change and statistical significance thresholds. Re-analysis using the DESeq2 framework confirmed robust transcriptional changes (Supplementary Table S1), including strong induction of interferon-stimulated genes and modulation of inflammatory mediators (Figure S1), consistent with activation of canonical innate immune pathways. Collectively, these results establish that stimulation elicits broad and robust transcriptional reorganization in human dendritic cells.

### 3.2. Differential Temporal Regulation of Antigen Presentation, Interferon, and Inflammatory Gene Modules

**Figure 2:**
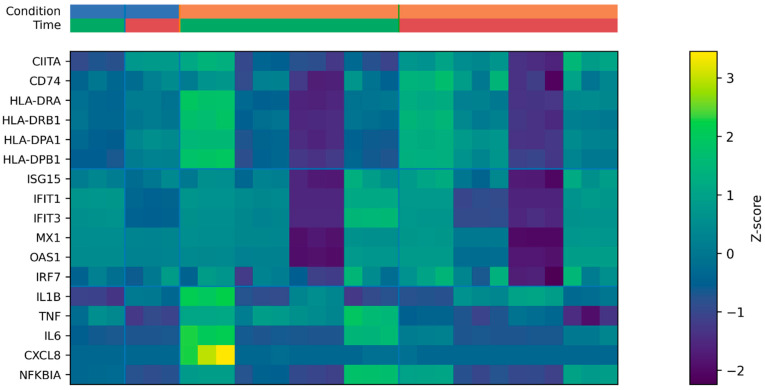
Differential expression patterns of antigen presentation, interferon, and inflammatory gene modules in human dendritic cells. Heatmap depicting the expression patterns of selected immune-related genes in human monocyte-derived dendritic cells from dataset GSE108526. Shown are genes involved in antigen presentation and MHC class II regulation (CIITA, CD74, HLA-DRA, HLA-DRB1, HLA-DPA1, HLA-DPB1), interferon-stimulated antiviral responses (ISG15, IFIT1, IFIT3, MX1, OAS1, IRF7), and inflammatory signaling (IL1B, TNF, IL6, CXCL8, NFKBIA). Expression values are shown as gene-wise Z-scores calculated from log_2_-transformed TPM values, enabling comparison of relative expression changes across samples. Columns represent individual samples and are ordered by condition and time point (Control 6 h, Control 16 h, Stimulated 6 h, Stimulated 16 h). Horizontal separators delineate the three predefined gene modules, while vertical separators indicate transitions between experimental groups. The top annotation bars indicate experimental metadata: condition (blue, Control; orange, Stimulated) and time point (green, 6 h; red, 16 h). Color intensity within the heatmap reflects relative expression levels, with red indicating higher-than-average expression and blue indicating lower-than-average expression for each gene. Together, the heatmap illustrates distinct and temporally structured transcriptional responses across antigen presentation, interferon, and inflammatory pathways following innate immune stimulation.

To resolve pathway-level dynamics, we examined expression patterns of predefined immune gene modules encompassing antigen presentation (MHC-II pathway), interferon-stimulated genes (ISGs), and inflammatory cytokines (Figure 2).

Heatmap visualization revealed consistent upregulation of ISGs following stimulation, particularly at 6 h, including ISG15, IFIT1, IFIT3, MX1, OAS1, and IRF7. This early and coherent ISG induction is consistent with activation of type I interferon signaling cascades.

Genes associated with antigen presentation and MHC class II regulation (CIITA, CD74, HLA-DRA, HLA-DRB1, HLA-DPA1, HLA-DPB1) demonstrated a distinct temporal pattern. Rather than immediate early induction, several components exhibited progressive modulation over time, suggesting a delayed or temporally distinct activation of antigen-processing machinery relative to early interferon responses.

The inflammatory module (IL1B, TNF, IL6, CXCL8, NFKBIA) showed heterogeneous but reproducible changes in expression, reflecting engagement of NF-κB-dependent transcriptional programs.

To evaluate the internal consistency of the predefined immune gene modules, we performed within-module co-expression analysis using Pearson correlation across all analyzed samples (Supplementary Figure S2). Genes within the MHC-II and ISG modules showed strong positive correlations, supporting coherent transcriptional behavior within these predefined sets. In contrast, the inflammatory module displayed more heterogeneous but still reproducible correlation patterns, consistent with the known variability of inflammatory gene regulation.

Together, these findings indicate that interferon, inflammatory, and antigen-presentation pathways are activated in a temporally structured and partially distinct manner rather than uniformly.

### 3.3. Quantitative Assessment of Module-Level Expression Dynamics

**Figure 3:**
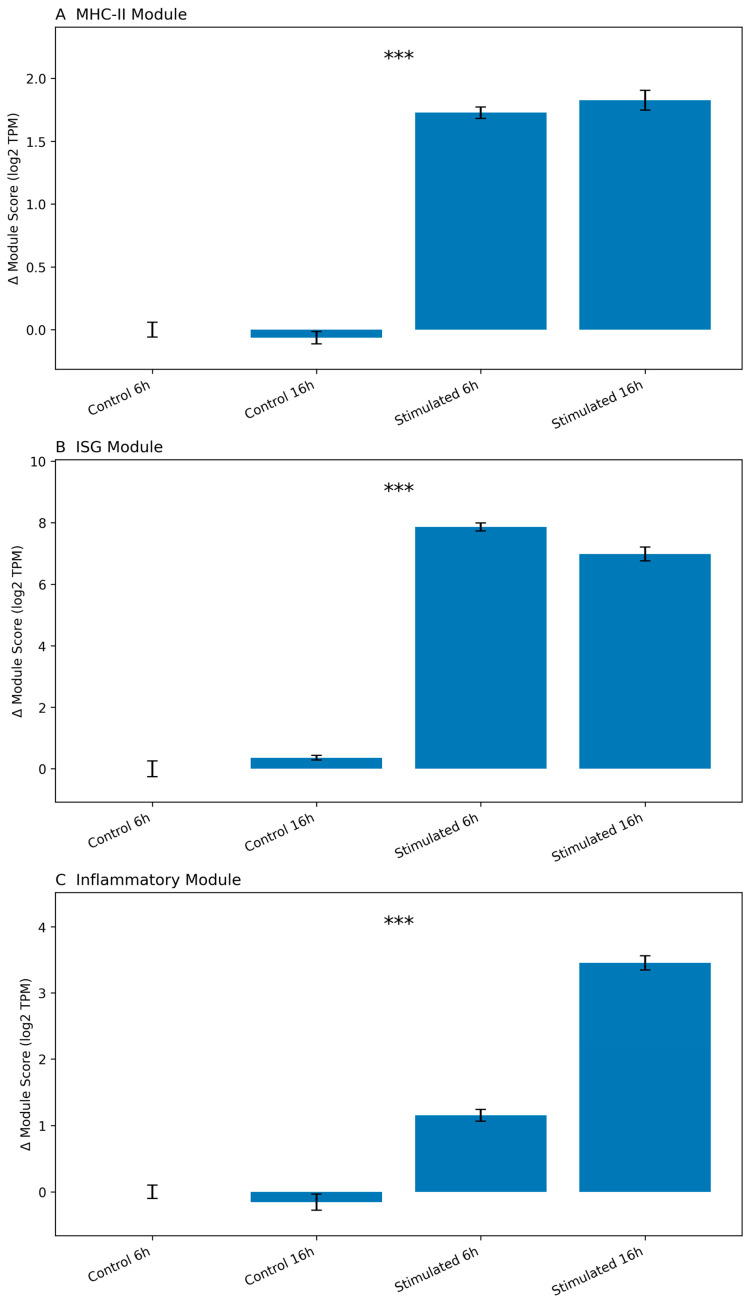
Quantification of immune gene module expression dynamics following stimulation. Module-level expression changes were quantified for three biologically defined gene sets: (**A**) antigen-presentation/MHC-II module (CIITA, CD74, HLA-DRA, HLA-DRB1, HLA-DPA1, HLA-DPB1), (**B**) interferon-stimulated gene (ISG) module (ISG15, IFIT1, IFIT3, MX1, OAS1, IRF7), and (**C**) inflammatory cytokine module (IL1B, TNF, IL6, CXCL8, NFKBIA). For each sample, module expression was calculated as the mean log_2_-transformed TPM value [log_2_(TPM + 1)] across all genes in the respective module. To facilitate comparison across conditions, module scores are shown as Δ values relative to the Control 6 h group, which was set as the reference baseline. Bars represent group means, and error bars indicate the standard error of the mean (SEM). The dashed horizontal line denotes the zero-baseline corresponding to Control 6 h. Results are shown for control and stimulated cells at 6 h and 16 h time points. Statistical significance was assessed using two-way ANOVA. Asterisks indicate the main effect of stimulation (condition) (*** *p* < 0.001).

To formally quantify pathway-level regulation, we computed module expression scores defined as the mean log_2_(TPM + 1) across genes within each module and expressed these values relative to the Control 6 h baseline (Δ values) (Figure 3).

Stimulation resulted in a clear increase in ISG module activity, particularly at the early 6 h time point, consistent with rapid engagement of the interferon pathway.

This response was maintained or modulated at 16 h, indicating sustained antiviral transcriptional programming.

The inflammatory module displayed modest but measurable induction relative to baseline. While less pronounced than ISG activation, inflammatory gene expression demonstrated consistent directional regulation following stimulation.

In contrast, the MHC-II module exhibited a distinct regulatory profile. Rather than immediate early induction comparable to ISGs, antigen presentation-related genes showed a more gradual and condition-dependent pattern of modulation.

Statistical evaluation using two-way ANOVA revealed a strong effect of stimulation (condition) across all modules (*p* < 0.001). The ISG module showed the largest magnitude of response, consistent with dominant interferon signaling. Inflammatory module scores demonstrated significant effects of both condition and time, indicating dynamic temporal modulation. In contrast, the MHC-II module was primarily influenced by stimulation status, with comparatively limited time-dependent variation.

This quantitative assessment confirms that interferon signaling represents the dominant early transcriptional program, whereas antigen presentation and inflammatory pathways exhibit distinct magnitudes and temporal kinetics.

### 3.4. Association Analysis Reveals Distinct Relationships Between Immune Gene Modules

**Figure 4:**
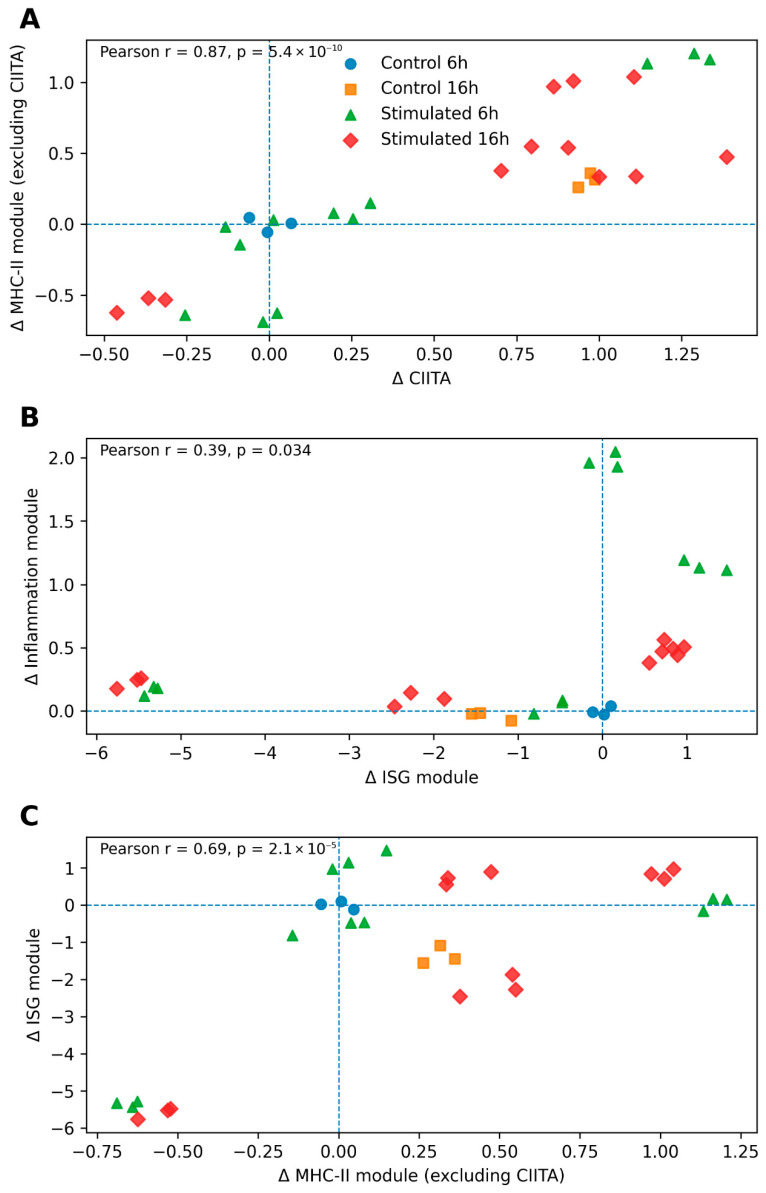
Associations between antigen presentation, interferon, and inflammatory transcriptional programs. Relationships between key immune transcriptional modules were examined using Δ-transformed expression values relative to the Control 6 h baseline. (**A**) Association between CIITA expression and the MHC-II module score (calculated excluding CIITA to avoid self-correlation), highlighting coordinated expression patterns within antigen-presentation genes. (**B**) Association between the interferon-stimulated gene (ISG) module and the inflammatory cytokine module, assessing the degree of concordant or divergent activation between antiviral and inflammatory responses. (**C**) Association between MHC-II module expression and ISG module activity, reflecting context-dependent relationships between antigen presentation and interferon-associated transcriptional responses. Each point represents an individual sample, with symbols indicating experimental condition and time point. Dashed horizontal and vertical lines denote the zero-baseline corresponding to Control 6 h. Pearson correlation coefficients (r) and associated *p*-values are shown for each panel. These analyses describe statistical associations and patterns of co-expression and do not imply direct regulatory or causal relationships between pathways.

To examine relationships between immune transcriptional programs, we performed association analyses with Δ-transformed expression values (Figure 4).

A strong positive association was observed between CIITA expression and the MHC-II module (excluding CIITA), consistent with coordinated expression within the antigen presentation pathway (Figure 4A). While this strong positive correlation supports the central role of CIITA in regulating MHC class II gene expression, the analysis reflects statistical association rather than direct regulatory causation.

Analysis of interferon and inflammatory modules revealed a positive but variable association (Figure 4B), suggesting that while these programs are co-activated, their magnitudes are not strictly proportional across samples. This finding is consistent with partially independent regulation of antiviral and inflammatory transcriptional responses.

Finally, examination of relationships between the MHC-II and ISG modules demonstrated context-dependent association (Figure 4C). While samples with stronger interferon activation tended to exhibit elevated antigen-presentation gene expression, the relationship was not uniformly linear, indicating differential and context-dependent transcriptional responses rather than a strictly hierarchical relationship.

Collectively, these analyses demonstrate that innate immune stimulation is associated with structured transcriptional responses across interferon, inflammatory, and antigen-presentation pathways, while these programs maintain distinct and non-uniform expression patterns across conditions and time points.

## 4. Discussion

Innate immune stimulation of dendritic cells initiates structured but non-uniform transcriptional programs that govern antiviral defense, inflammatory signaling, and antigen presentation. In the present in silico re-analysis of GSE108526, we demonstrate that interferon-stimulated gene (ISG) activation represents the dominant early transcriptional axis, whereas inflammatory cytokine induction and MHC class II-related antigen presentation programs display distinct magnitudes and temporal kinetics. Importantly, association-based analysis reveals that these pathways, while interconnected, are not strictly proportional, highlighting modular and partially independent transcriptional regulation.

Our global variance analysis and module quantification demonstrate that interferon-driven gene expression constitutes the principal source of transcriptomic separation following stimulation. This observation is consistent with established models in which type I interferon responses are rapidly amplified through IRF-dependent feed-forward loops and JAK–STAT signaling cascades [18,19]. ISGs such as ISG15, IFIT family members, and MX1 are among the earliest transcriptional responders to viral and nucleic acid stimuli and function not only in antiviral restriction but also in shaping cellular metabolic state and antigen-processing capacity [20,21].

Recent systems immunology studies indicate that interferon signaling can impose a dominant transcriptional “state” that persists beyond the initiating stimulus and influences the expression landscape of secondary pathways [22]. This may explain the sample clustering observed in the PCA and the robust Δ module elevation in our ISG analysis. Importantly, interferon-dominant states have been associated with enhanced cross-presentation, modulation of co-stimulatory molecules, and altered DC survival dynamics, suggesting that early IFN signaling establishes a regulatory scaffold upon which later adaptive functions are layered [23].

Although inflammatory cytokine genes were induced following stimulation, their magnitude and association with the ISG module were less uniform. NF-κB activation is necessary but not sufficient for maximal cytokine transcription; chromatin accessibility, enhancer priming, and interplay with IRF family members influence gene-specific magnitude and duration [24,25]. Recent work demonstrates that transcriptional burst kinetics of inflammatory genes can vary markedly even within the same signaling context, contributing to heterogeneous inflammatory outputs [26].

Our association analysis between ISG and inflammatory modules revealed a positive but non-linear relationship, consistent with partial independence between antiviral and inflammatory axes. This aligns with emerging evidence that interferon signaling can attenuate excessive inflammatory cytokine production via negative feedback regulators such as SOCS proteins and interferon-induced suppressors [27]. Thus, rather than functioning as a single unified activation program, interferon and inflammatory transcriptional modules exhibit coordinated but constrained interdependence.

A central question addressed in this study is the relationship between CIITA-dependent antigen presentation and innate antiviral signaling. While interferons can promote MHC-II transcription indirectly, the kinetics of CIITA induction often differ from immediate early ISG responses [28]. Our Δ module analysis indicates that MHC-II-associated genes display more gradual modulation relative to ISGs.

Importantly, by recalculating the MHC-II module excluding CIITA, we demonstrate that CIITA expression is positively associated with downstream MHC-II gene expression, supporting its role as a transcriptional coordinator. However, the magnitude of this relationship does not perfectly mirror ISG induction, suggesting that antigen presentation programs are not a simple derivative of interferon activity. This observation is consistent with epigenetic studies showing promoter-specific chromatin remodeling requirements for MHC-II genes that introduce regulatory delays or stimulus-specific constraints [29,30].

Additionally, antigen presentation pathways can be influenced by metabolic state, autophagic flux, and endosomal processing dynamics, all of which are modulated by innate immune activation but not strictly governed by interferon amplitude [31]. These additional layers of regulation likely contribute to the structured yet non-identical relationships observed between MHC-II and ISG modules.

Rather than identifying activation of individual pathways in isolation, this study provides a quantitative comparison of major immune transcriptional modules, highlighting differences in magnitude, temporal dynamics, and inter-module relationships. Our results support a model in which DC activation is organized into semi-autonomous transcriptional modules rather than a monolithic maturation program. Systems-level reconstructions of pathogen-response networks have revealed that immune signaling pathways cluster into interconnected yet partially separable subnetworks that respond with different gain, kinetics, and feedback control [32,33]. The associations observed in our analysis—strong CIITA-to-MHC-II alignment, moderate ISG-to-inflammatory relationships, and context-dependent MHC-II-to-ISG patterns—fit this framework.

Such modular architecture may provide functional flexibility, enabling DCs to tailor antigen presentation capacity independently of inflammatory amplitude, or to sustain antiviral vigilance without proportionate inflammatory escalation. This separation could be particularly relevant in chronic viral exposure, autoimmune contexts, or vaccine adjuvant design where qualitative rather than purely quantitative immune outputs determine outcome [34].

## 5. Limitations and Future Directions

This study is limited by reliance on bulk RNA-seq data and predefined gene modules. While module scoring provides biologically interpretable pathway-level quantification, future analyses incorporating gene set enrichment approaches (e.g., GSVA) or single-cell transcriptomic validation would further refine observed inter-module associations. Additionally, mechanistic validation of CIITA kinetics and chromatin accessibility dynamics would strengthen interpretation of transcriptional relationships.

Nevertheless, by combining global variance analysis, DESeq2-based differential expression, module-based quantification, and inter-module association analysis, this study provides a structured framework for dissecting transcriptional architecture in DC activation.

## 6. Conclusions

In summary, innate immune stimulation of human dendritic cells produces a transcriptional landscape dominated by early interferon signaling, and is accompanied by context-dependent inflammatory activation and differentially regulated antigen presentation programs. These pathways exhibit coordinated but partially independent relationships, revealing modular transcriptional organization. Our reproducible analytical framework highlights the value of quantitative module-level and association-based analyses for understanding structured immune activation in human dendritic cells.

## Data Availability

All transcriptomic analyses in this study were performed using publicly available RNA-sequencing data from the Gene Expression Omnibus (GEO) under accession number GSE108526. All scripts used for data preprocessing, gene annotation mapping (GeneID to gene symbol), differential expression analysis, gene-set scoring, module quantification, coupling and correlation analyses, and figure generation are provided as a versioned supplementary compressed archive accompanying this manuscript. This supplementary reproducibility package includes fully annotated Python and R scripts and documentation enabling complete reproduction of all analyses and figures presented in this study.

## Supplementary Materials

The following supporting information can be downloaded at: https://www.mdpi.com/article/10.3390/biology15080636/s1, Supplementary Figure S1: DESeq2-based differential expression validation; Supplementary Figure S2: Within module coexpression; Supplementary Table S1: DESeq2_results; Supplementary Data S1: Reproducibility and computational package.
