# Supplementary material for: CIITA-Linked Antigen Presentation Is Differentially Associated with Interferon and Inflammatory Programs in Stimulated Human Dendritic Cells
Source: Biology (Basel). 2026 Apr 17;15(8):636. doi: 10.3390/biology15080636 (PMC13114228; doi:10.3390/biology15080636)
Supplement: Supplementary file 1 [file biology-15-00636-s001.zip › Supplementary Figure S1 DESeq2-based DE validation with Legend.pdf]

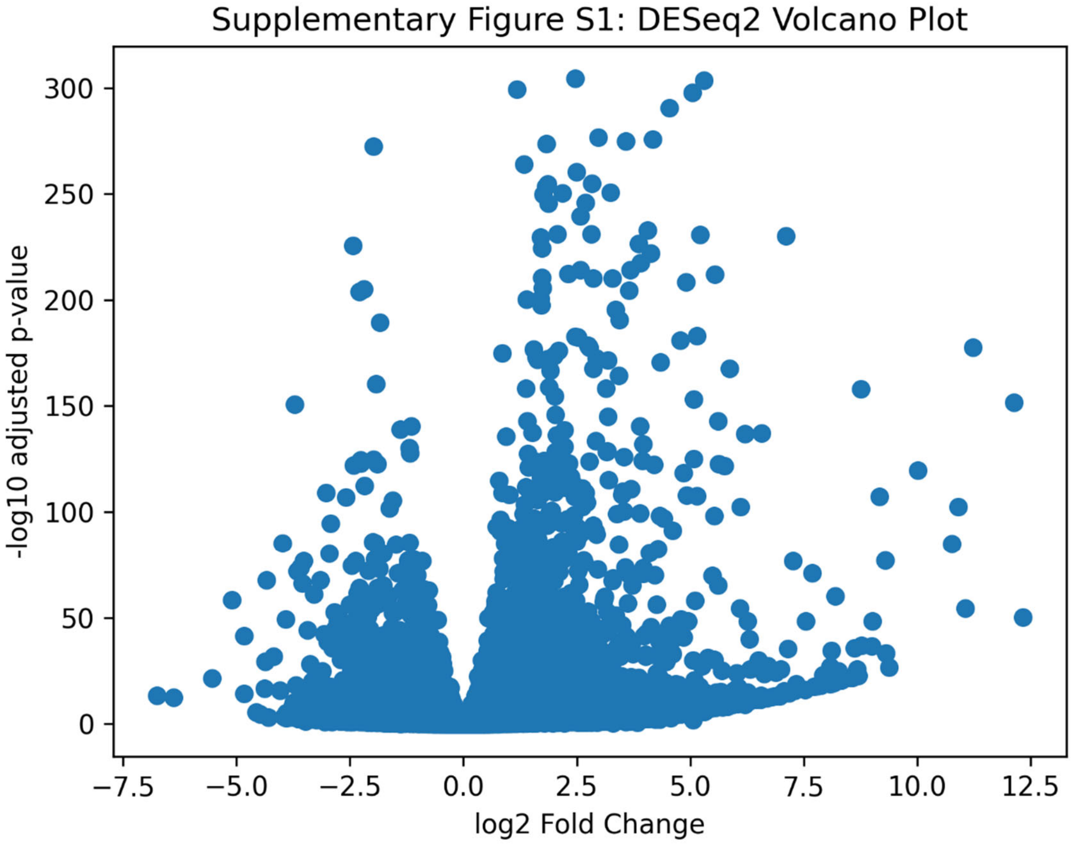

Supplementary Figure S1. DESeq2-based differential expression analysis of stimulated versus control dendritic cells. Volcano plot showing genome-wide differential gene expression between stimulated and control human dendritic cells based on DESeq2 analysis of raw count data. The x-axis represents $\log_2$ fold change (stimulated vs control), and the y-axis represents –$\log_{10}$ adjusted p-value (FDR). Each point corresponds to a single gene. Genes meeting the predefined significance thresholds ($|\log_2$ fold change$| \geq 1$ and adjusted p-value (FDR) < 0.05) are considered differentially expressed. The distribution of points illustrates widespread transcriptional remodeling following stimulation, with both upregulated and downregulated genes observed across the transcriptome.
