# Supplementary material for: CIITA-Linked Antigen Presentation Is Differentially Associated with Interferon and Inflammatory Programs in Stimulated Human Dendritic Cells
Source: Biology (Basel). 2026 Apr 17;15(8):636. doi: 10.3390/biology15080636 (PMC13114228; doi:10.3390/biology15080636)
Supplement: Supplementary file 1 [file biology-15-00636-s001.zip › Supplementary Figure S2 within_module_coexpression with Legend.pdf]

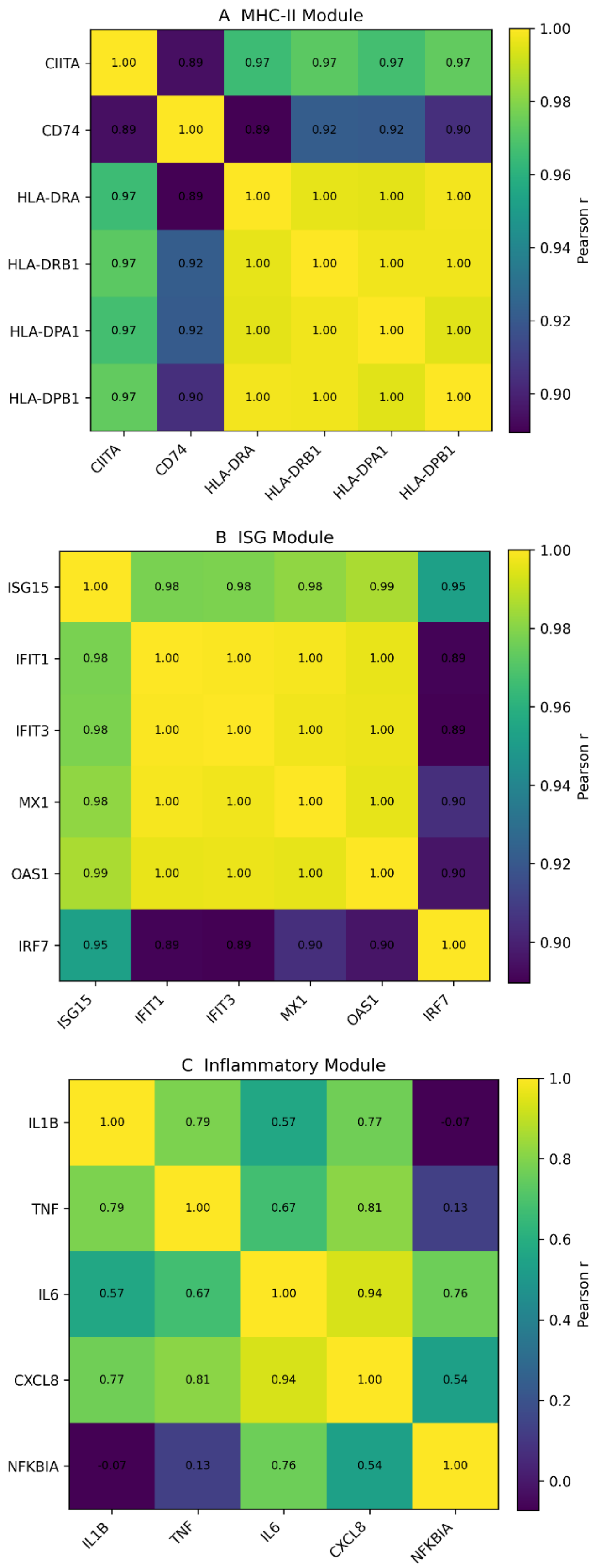
A MHC-II Module
CIITA
CD74
HLA-DRA
HLA-DRB1
HLA-DPA1
HLA-DPB1
1.00 0.89 0.97 0.97 0.97 0.97
0.89 1.00 0.89 0.92 0.92 0.90
0.97 0.89 1.00 1.00 1.00 1.00
0.97 0.92 1.00 1.00 1.00 1.00
0.97 0.92 1.00 1.00 1.00 1.00
0.97 0.90 1.00 1.00 1.00 1.00
Pearson r
1.00
0.98
0.96
0.94
0.92
0.90
B ISG Module
ISG15
IFIT1
IFIT3
MX1
OAS1
IRF7
1.00 0.98 0.98 0.98 0.99 0.95
0.98 1.00 1.00 1.00 1.00 0.89
0.98 1.00 1.00 1.00 1.00 0.89
0.98 1.00 1.00 1.00 1.00 0.90
0.99 1.00 1.00 1.00 1.00 0.90
0.95 0.89 0.89 0.90 0.90 1.00
Pearson r
1.00
0.98
0.96
0.94
0.92
0.90
C Inflammatory Module
IL1B
TNF
IL6
CXCL8
NFKBIA
1.00 0.79 0.57 0.77 -0.07
0.79 1.00 0.67 0.81 0.13
0.57 0.67 1.00 0.94 0.76
0.77 0.81 0.94 1.00 0.54
-0.07 0.13 0.76 0.54 1.00
Pearson r
1.0
0.8
0.6
0.4
0.2
0.0

Supplementary Figure S2. Within-module co-expression analysis of predefined immune gene modules. Pearson correlation heatmaps showing pairwise relationships among genes within the (A) MHC-II, (B) interferon-stimulated gene (ISG), and (C) inflammatory modules across the analyzed samples. Correlation coefficients are shown within each cell. The strong positive correlations observed within the MHC-II and ISG modules support the internal consistency of the predefined gene sets, while the inflammatory module shows more heterogeneous but reproducible co-expression patterns.
